# Supplementary material for: Identification of photoperiod-induced specific miRNAs in the adrenal glands of Sunite sheep (Ovis aries)
Source: Front Vet Sci. 2022 Jul 22;9:888207. doi: 10.3389/fvets.2022.888207 (PMC9354845; doi:10.3389/fvets.2022.888207)
Supplement: Supplementary file 1 [file Data_Sheet_1.ZIP › Supplementary file/miRDeep2 file.zip!/miRDeep2 file/Novel_Structure/SPLP42A1/Novel_430.pdf]

Provisional ID	:	NC_019479.2_27791
Score total	:	1.8
Score for star read(s)	:	-1.3
Score for read counts	:	0
Score for mfe	:	1.5
Score for randfold	:	1.6
Score for cons. seed	:	
Total read count	:	94
Mature read count	:	94
Loop read count	:	0
Star read count	:	0

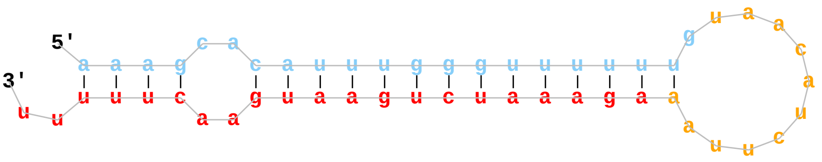

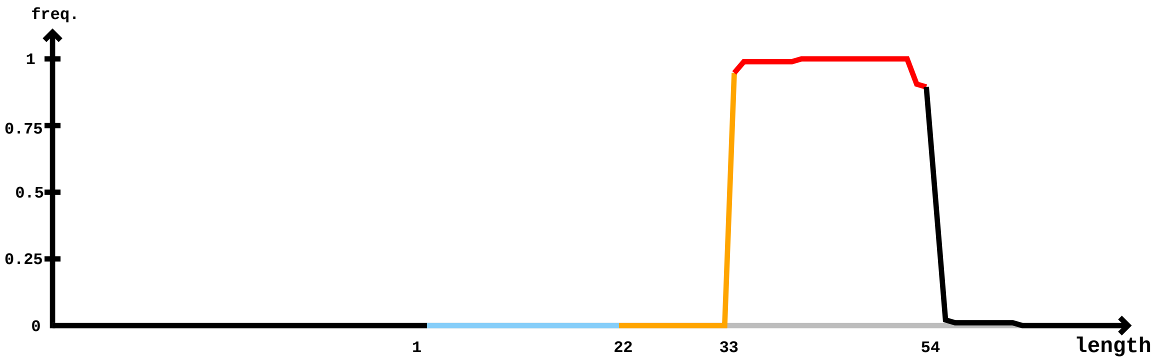


```
                                           Star                                 Mature
5'- accaaaauuacagaggcuaauuuauugguuggcuaaaaagcacauuuggguuuuuuguaacaucuuaaagaaaucugaaugaacuuuuugggcaacccaauacauucua   -3'    exp
    ......................((((((((((.((((((((..((((((((((((((..........))))))))))))))..)))))))).)))))))))).......    reads      mm         sample
    ....................................................................aAaaaucugaaugaacuuu......................    9          1          seq
    ....................................................................agaaaCcugaaugaacuuuu.....................    1          1          seq
    ....................................................................aAaaaucugaaugaacuuuuu....................    80         1          seq
    .....................................................................gaaaucugaaugaacuuuuu....................    2          0          seq
    .....................................................................gaaaCcugaaugaacuuuuu....................    1          1          seq
    .....................................................................gaaaucugaaugaacuuuuugg..................    1          0          seq
    ...........................................................................ugaaugaacuuuuuggUcaaccc...........    1          1          seq
```
